# Advanced Nanoscale Materials and (Flexible) Devices

**DOI:** 10.3390/nano15211662

**Published:** 2025-11-01

**Authors:** Kaiwen Lin, Chunhui Duan, Baoyang Lu

**Affiliations:** 1 Department of Materials and Food, University of Electronic Science and Technology of China Zhongshan Institute, Zhongshan 528402, China; 2 Institute of Polymer Optoelectronic Materials and Devices, State Key Laboratory of Luminescent Materials and Devices, South China University of Technology, Guangzhou 510640, China; 3 Jiangxi Provincial Key Laboratory of Flexible Electronics, School of Pharmacy, Jiangxi Science & Technology Normal University, Nanchang 330013, China

## 1. Introduction

The fusion of nanoscience and mechanically compliant systems is redefining the performance boundaries of electronics, photonics, energy, and sensing technologies [1,2]. Over the past two decades, the research field has witnessed a transition from rigid bulk semiconductors to ultrathin, low-dimensional materials [3,4]. Meanwhile, the urgent demand for wearable, implantable, and Internet of Things (IoT) hardware has further accelerated the shift to flexible, stretchable, and even self-healing forms [5,6]. The resulting research direction of “advanced nanoscale materials and (flexible) devices” has become a frontier where chemistry, physics, materials chemistry, and information engineering intersect.

Advanced nanoscale materials and (flexible) devices represent the cutting-edge intersection of multiple disciplines, including materials science, nanotechnology, electronics, physics, chemistry, and engineering. This field is committed to precisely constructing new materials and devices at the nanoscale, endowing them with mechanical properties such as bendability, stretchability, and adaptability [7,8]. Through continuous innovation in fabrication processes and performance tuning strategies, researchers are constantly pushing the boundaries of functionality. This progress is driving paradigm shifts in industries such as electronic healthcare, energy and the environment, and intelligent manufacturing, infusing core momentum into future technological revolutions [9,10].

This Special Issue gathers cutting-edge research on the above-mentioned topics. The collected papers provide readers with profound insights from the perspectives of material aspects, phase transitions, and device performance, helping to transform nanoscale material breakthroughs into next-generation flexible technologies that are high-performance, durable, and sustainable.

## 2. An Overview of Published Articles

This Special Issue collates eight articles, specifically six research articles and two review articles, dedicated to advanced nanoscale materials and (flexible) devices. The overview of published articles in the Special Issue includes the following.

Cai et al. innovatively proposed a design strategy to extend the length of the source–drain epitaxial region (L_ext_) under a vertically stacked architecture, which maintains a high drive current and suppresses the quantum tunneling effect on the drain side. For the sidewall gate architecture, single-walled carbon nanotubes were employed as the channel material can significantly reduce the off-state current (I_off_ = 10^−14^ A) [11].

Tang et al. enabled simultaneous defect passivation and crystallization control for high-efficiency inverted perovskite solar cells via bifunctional 4,5-diiodoimidazole interfacial engineering, which achieved a power conversion efficiency (PCE) of 24.10%, with a V_OC_ enhancement from 1.12 to 1.14 V, and maintained 91% of initial PCE after 1300 h [12].

Tang et al. addressed the critical challenges of interfacial defects and insufficient stability in perovskite solar cells by introducing a co-solvent engineering strategy to dynamically regulate the phenethylammonium chloride passivation layer, enabling the oriented growth of a dense layer, yielding a champion power conversion efficiency of 24.27% [13].

Li et al. improved the device performance on a fully transparent amorphous InGaZnO_4_ (a-IGZO) top-gate thin-film transistor (TG-TFT) via a two-step annealing treatment. The optimized best performance presented a mobility of 13.05 cm^2^/(V·s), a threshold voltage (Vth) of 0.33 V, a subthreshold swing of 130 mV/dec, and an I_on_/I_off_ of 1.73 × 10^8^ with a transmittance of over 90% [14].

Musin et al. studied the relationship between the size factor and the dopant concentration range for the formation and stabilization of the cubic phase in scandium-stabilized zirconia (ScSZ) films. The crystal structure of films with an average crystallite size of 8.5 nm was cubic at Sc_2_O_3_ content from 6.5 to 17.5 mol%, which is much broader than the range of 8–12 mol% of the conventional deposition methods. The sputtering of ScSZ films on hot substrates resulted in a doubling of crystallite size and a decrease in the cubic phase range to 7.4–11 mol% of Sc_2_O_3_ content [15].

Zhang et al. studied the nano-structure evolution and mechanical properties of Al_x_CoCrFeNi_2.1_ (x = 0, 0.3, 0.7, 1.0, 1.3) high-entropy alloy prepared by mechanical alloying and spark plasma sintering. With the increasing Al content, the crystal structure of the alloys gradually transformed from a nanocrystalline phase of FCC to a mix of FCC and BCC nanocrystalline. Meanwhile, the hardness was found to increase steadily from 433 HV to 565 HV due to the increase in the fraction of BCC nanocrystalline phase [16].

Wan et al. discussed recent progress in neuromorphic visual perception based on ferroelectric materials, elaborating in detail on device structure, material systems, and applications, and exploring the potential future development trends and challenges faced in the human-like visual perception field [17].

Liu et al. presented an in-depth review of the evolution of hydrogels applied for fire extinguishing and prevention. The review explained the extinguishing principles of hydrogel extinguishants, discussed the preparation strategies and evaluation system of the hydrogel extinguishants, and highlighted the importance of considering the commercial aspects of hydrogel extinguishants [18].

## 3. Conclusions

Our Special Issue may promote and accelerate ongoing research efforts of advanced nanoscale materials and (flexible) devices; meanwhile, we have carried out the collection work for the second volume on the same theme. It is of vital importance to the synthesis of advanced materials, the preparation of (flexible) devices, the engineering of the nanophase, and the application of advanced materials and (flexible) devices, which will be of interest to general readers of *Nanomaterials*.
